# Patterns of alcohol consumption in european pregnant women with alcohol use disorder

**DOI:** 10.1192/j.eurpsy.2021.1487

**Published:** 2021-08-13

**Authors:** I. Pereira, V. Nogueira, J. Teixeira

**Affiliations:** Clínica 4 - Unidade De Alcoologia E Novas Dependências, Centro Hospitalar Psiquiátrico de Lisboa, Lisboa, Portugal

**Keywords:** alcohol consumption patterns, pregnancy, alcohol use disorder

## Abstract

**Introduction:**

Prenatal alcohol exposure can have a negative impact on a child’s neurocognitive development. Still, about 16% of European women maintain alcohol consumption, even after knowing they are pregnant. Several studies have shown that alcohol use patterns alter drastically during pregnancy. However, little is known about how these change in women with Alcohol Use Disorder (AUD) diagnosis.

**Objectives:**

To understand the impact of pregnancy on alcohol use patterns in women at high risk or with previous AUD diagnosis.

**Methods:**

Bibliographic research was made through the PubMed/NCBI database. No time limit was specified on the search. Pertinent manuscripts were individually reviewed for additional relevant citations.

**Results:**

Several factors influence alcohol consumption during pregnancy, including financial status, educational level, and high levels of psychological stress. Although older age at the onset of pregnancy is deemed a risk factor for alcohol consumption, women of 25 or fewer years of age are at higher risk for AUD, as are those with a history of criminal behaviour and family history of AUD. Pregnancy seems to play a critical role in altering alcohol use patterns, reducing the risk of AUD in about 70%, regardless of pregnancy trimester. This is seen even in women who present high-risk factors for AUD.

**Conclusions:**

Pregnancy presents itself as a behavioural change promoter and should be regarded as a window of opportunity for intervention in women with AUD. However, there are few studies that focus on alcohol consumption patterns specifically in women with AUD, whereby making it necessary to extrapolate the available data.

